# Editorial: Neuronal Self-Defense: Compensatory Mechanisms in Neurodegenerative Disorders

**DOI:** 10.3389/fncel.2015.00499

**Published:** 2016-01-05

**Authors:** Rosanna Parlato, Pier G. Mastroberardino

**Affiliations:** ^1^Institute of Applied Physiology, University of UlmUlm, Germany; ^2^Institute of Anatomy and Medical Cell Biology, University of HeidelbergHeidelberg, Germany; ^3^Department of Genetics, Erasmus Medical CenterRotterdam, Netherlands

**Keywords:** neurodegenerative disorders, mechanisms, stress responses, compensation, models, molecular

Neurodegenerative diseases (ND) are characterized by the progressive loss of specific neuronal populations with consequent deterioration of brain's function. While some ND have monogenic causes, the vast majority is sporadic. Accordingly, etiology varies greatly between these conditions; the pathogenesis, however, shares important common traits. Trophic deprivation, oxidative stress, accumulation of abnormal protein aggregates, and bioenergetics defects have been in fact described in most, if not all, ND. To counterbalance these noxious stimuli cells deploy intrinsic neuroprotective responses, at least during early pathogenesis. Adaptation includes strategies to optimize energetic resources, for instance reduction of rRNA synthesis to repress translation, suppression of transcription, and bioenergetics and metabolic redesign. Additional mechanisms include potentiation of antioxidant capacity, induction of endoplasmic reticulum (ER) stress, and activation of protein quality control systems and autophagy. Strategies to potentiate these naturally occurring processes might provide foundation to devise new experimental treatments.

This e-book contains a collection of reviews and original articles summarizing the state-of-the-art knowledge on protective responses sustaining neuronal survival and activity. The ultimate goal of this work is to inspire novel studies elucidating strategies to contrast these incurable disorders.

The first article reviews transcriptional deregulation in Huntington's disease (HD). Francelle and colleagues point out that while transcriptional changes are directly caused by mutant huntingtin, they may also represent a response to secondary stress conditions triggered by dysfunctional transcriptional machineries. They speculate that transient expression changes typical of HD may represent a self-defense mechanism because some of these altered genes activate pro-survival functions, which may be impaired during aging. Hence, the investigation of gene expression changes with aging in the striatum, the brain region mostly affected in HD, might help to develop strategies to modify mutant huntingtin toxicity (Francelle et al.). In line with this concept, Stilling and colleagues show how aging is associated with changes in gene expression and in RNA splicing, and with the upregulation of immune system functions in the hippocampus (Stilling et al.). Interestingly, affected genes are involved in synaptic function, therefore suggesting a potential pathogenic link between age-dependent transcriptional alterations and late onset of Alzheimer's disease (AD) (Stilling et al.).

A second original research article illustrates how two transcription factors important for the development of dopaminergic neurons also play a role in their maintenance in adulthood. Previous studies indicated that haploinsufficiency of the transcription factor Foxa 2 leads to abnormalities in motor behavior in old age and an associated progressive loss of dopaminergic neurons reminiscent of Parkinson's disease (PD) (Kittappa et al., 2007). Here Domanskyi and colleagues report that Foxa1 and Foxa2 co-evolved to compensate the lack of each other. By the generation and analysis of specific conditional double mutant mice they show the protective role of these transcription factors for the maintenance of dopaminergic neurons in adult stages (Domanskyi et al.).

A third original article addresses neuronal self defense mechanisms based on the regulation of rRNA synthesis. Downregulation of rRNA synthesis has been associated with neuronal loss in several ND, although disruption of nucleolar activity may trigger context-specific neuroprotective responses (Kiryk et al.; Kreiner et al., 2013). The study by Riancho and colleagues reveals that active nucleolar transcription may be a compensatory mechanism in a mouse model of amyotrophic lateral sclerosis (ALS) at both asymptomatic and symptomatic stages. High rate of rRNA transcription in ALS motor neurons could maintain protein synthesis when proteostasis is severely impaired (Riancho et al.).

Edwin Chan reviews aberrant RNA species—an emerging mechanism of toxicity in ND—particularly in polyQ diseases linked to nucleotide repeat expansion and in frontotemporal dementia/ALS (FTD/ALS). The piece discusses the impact of these mutant RNAs and proposes that toxicity may be ascribed to alternative splicing of RNAs, hairpin, and double-stranded CAG repeat RNAs, sequestration of cellular proteins, nucleolar stress, repeat associated non-ATG translation (RAN-translation) of CAG expansion transcripts (Chan).

The role of antisense long noncoding RNAs (lncRNAs) as regulators of neuronal gene expression is rapidly emerging (Ng et al., 2013). Recent studies indicate that they could contribute to ND. This mechanism has been for instance demonstrated in mouse, for the ubiquitin carboxy-terminal hydrolase L1 (Uchl1). Importantly in human UCHL1 (or PARK5) has been associated with familial PD. Carrieri and colleagues propose that increasing its expression might be beneficial and antisense (AS) Uchl 1 could be used to increase Uchl1 mRNA translation. They also show that AS Uchl 1 promoter region contains a binding site for Nurr1, a transcription factor required for differentiation of dopaminergic neurons. Moreover, AS Uchl 1 level is reduced in the prototypical MPTP model of PD, suggesting that this novel stress mechanism responding to stress might have a pathological relevance in PD pathophysiology (Carrieri et al.). In the context of this post-transcriptional regulation of protein synthesis, this group also proposes in a second original article the use of antisense long noncoding RNAs in neuronal cell lines to activate translation in a gene specific manner (Zucchelli et al.). Finally, Amadio and colleagues contributed with another original article on post-transcriptional regulation, proposing that ELAV (embryonic lethal abnormal vision) proteins to heme-oxygenase-1 mRNA—a gene activated to counteract oxidative stress—to increase its expression and elicit neuroprotection with beneficial effects (Amadio et al.).

After this initial series of articles focusing on transcriptional and post-transcriptional regulation, the collection continues with articles discussing compensatory mechanisms based on translation suppression.

The mammalian (or mechanistic) target of rapamycin (mTOR) acts as a central regulator of cell homeostasis, and its activity is tightly regulated (Laplante and Sabatini, 2012). mTOR plays an important role in neuronal homeostasis (Bateup et al.). In this context the minireview by Canal and colleagues focuses on the mTOR inhibitor RTP801/REDD1, a protein encoded by the stress responsive gene DNA damage inducible transcript 4. REDD1 expression is regulated during development and in response to DNA damage. In addition, REDD1 is induced by stressors, such as hypoxia and ER stress. Interestingly REDD1 is also found upregulated in PD. The authors propose a model in which REDD1 may have either a protective or toxic function in cell survival depending on its protein level that changes at different disease stages. At low level, REDD1 might be a protective compensatory response at early disease stage (Canal et al.).

The next group of articles deals with accumulation of protein aggregates, which constitutes another shared mechanism of neurodegeneration and represents a promising therapeutic target. The review by Angelika and Fabio Falsone focuses on different regulators of protein aggregates, including stress granules (SG) (Falsone and Falsone). SG are dynamic cellular compartments that contribute to the arrest of mRNA translation in response to stress insults, such as UV-irradiation, oxidative stress. SG are also linked to amyloidogenic proteins and irreversible protein aggregates perturb their normal dynamics. Alteration of SG dynamics however may be due to irreversible protein aggregates. The articles also explores the nexus between heat shock proteins and proteolytic pathways, and specifically focuses on the crosstalk between different protein degradation pathways such as ubiquitin-proteasome system, chaperone mediated autophagy and macroautophagy. The work reveals that these mechanism share a certain degree of redundancy and the function of the unaffected one can rescue malfunction of the other pathways (Falsone and Falsone).

Jaronen and coworkers focus in their review on the role of ER stress and unfolded protein response (UPR) on the protein disulfide isomerase (PDI), a chaperone regulator of misfolded protein degradation, in ALS (Jaronen et al.). PDI is upregulated in ALS and this upregulation is shown early in the disease progression in ALS rat models. PDI inactivation increases ER stress, defined as disruption of the ER linked to accumulation of misfolded proteins, while at early stages PDI could prevent aggregation of superoxide dismutase 1 (SOD1). However, at a later stage increased UPR can result in increased PDI activation that may result in neurotoxicity by increasing superoxide production or according to a second model by increasing hydroperoxyde (Jaronen et al.). A third mini-review on this topic, contributed by Ujval Anilkumar and Jochen Prehn, discusses non-cell death related functions of anti-apoptotic proteins such as the pro-survival BCL-2 in mitochondrial physiology and ER homeostasis (Anilkumar and Prehn). J proteins, which belong to the chaperone family, may exert neuroprotective effects by facilitating proteostasis, as also discussed in the mini-review by Koutras and Braun.

Sugaya and Nagano propose an alternative hypothesis to explain the variability in the onset of familial ALS (fALS) despite same SOD1 genetic mutation. Based on available data for SOD1-linked fALS and a risk-based modeling approach, the authors suggest a SOD1 prion-like model of propagation. Spread of toxic aggregates through cell-to-cell transmission has been observed in several ND. However, the “protective aggregation hypothesis” according to which misfolded protein aggregates may represent a defensive response to protect cells from more toxic oligomeric species is hypothesized also for SOD1-linked fALS (Sugaya and Nakano). This article considers a “one-hit model,” i.e., a mathematical model in which the risk of cell death remains constant. The next review article by Fiebich and colleagues, instead, formulates a two-hit hypothesis that includes also neuroinflammation (Fiebich et al.). Anti-neuroinflammatory approaches represent a promising strategy against neurodegeneration and this review discusses the role of P2 receptors, which can bind ATP released by injured cells. This represents a second detrimental event synergizing with aggregation and the possibility of decreasing neuroinflammation by targeting P2 receptors is accordingly discussed (Fiebich et al.).

In the context of neuroprotective strategies aiming at potentiating the cell antioxidant capacity, the inhibition of NMDA receptor and the calcium dependent enzyme nitric oxide synthase (nNOS) activities is discussed by Courtney et al. In particular the authors review the role of NOS1AP, an adaptor protein which is considered a nNOS inhibitor. As a modulator of the excessive nNOS, NOS1AP may play a role in the neuronal self-defense against the NMDAR mediated excitotoxicity dependent on nNOS signaling (Courtney et al.).

Ca^2+^ overload is a pathogenic feature of sporadic and fALS, characterized by degeneration of motor neurons (MNs). In this original article by Mühling and colleagues, pharmacological stimulation of Ca^2+^ transporters in this vulnerable neuronal population is presented as a potential neuroprotective strategy. The authors show that, in SOD1G93A mice, mitochondrial and plasma membrane Ca^2+^ transporters are expressed at higher levels in MNs dissected from the hypoglossal nucleus. These findings are in line with an activity dependent Ca^2+^ clearance deficit and could represent a compensatory response to the disease (Mühling et al.).

In the last review closing this e-book, Grzegorz Kreiner elaborates on the difficulties of modeling ND and arguments that, despite the unavoidable differences between human and rodents, compensatory mechanisms shown in mouse models could help developing new hypotheses to devise neuroprotective strategies (Kreiner).

We hope that the present e-book provides a better understanding of mechanisms and their interconnections regulating neuronal homeostasis, and how ineffective execution of compensatory strategies severely threatens cellular homeostasis favoring pathogenesis. We believe that this comprehensive collection represents an original reference and may inspire innovative studies that will devise more effective therapeutic tools to prolong neuronal survival and activity.

## Funding

The support from the Deutsche Forschungsgemeinschaft (PA 1529/2-1) to RP is here acknowledged.

### Conflict of interest statement

The authors declare that the research was conducted in the absence of any commercial or financial relationships that could be construed as a potential conflict of interest.

## References

[B1] Kittappa R. Chang W. W. Awatramani R. B. McKay R. D. (2007). The foxa2 gene controls the birth and spontaneous degeneration of dopamine neurons in old age. PLoS Biol. 5:e325. 10.1371/journal.pbio.0050325 18076286 PMC2121110

[B2] Kreiner G. Bierhoff H. Armentano M. Rodriguez-Parkitna J. Sowodniok K. Naranjo J. R.. (2013). A neuroprotective phase precedes striatal degeneration upon nucleolar stress. Cell Death Differ. 20, 1455–1464. 10.1038/cdd.2013.66 23764776 PMC3792439

[B3] Laplante M. Sabatini D. M. (2012). mTOR signaling in growth control and disease. Cell 149, 274–293. 10.1016/j.cell.2012.03.017 22500797 PMC3331679

[B4] Ng S. Y. Lin L. Soh B. S. Stanton L. W. (2013). Long noncoding RNAs in development and disease of the central nervous system. Trends Genet. 29, 461–468. 10.1016/j.tig.2013.03.002 23562612

